# Plant Roots Release Small Extracellular Vesicles with Antifungal Activity

**DOI:** 10.3390/plants9121777

**Published:** 2020-12-15

**Authors:** Monica De Palma, Alfredo Ambrosone, Antonietta Leone, Pasquale Del Gaudio, Michelina Ruocco, Lilla Turiák, Ramesh Bokka, Immacolata Fiume, Marina Tucci, Gabriella Pocsfalvi

**Affiliations:** 1 Institute of Biosciences and BioResources, Research Division Portici, National Research Council, 80055 Portici, Italy; monica.depalma@ibbr.cnr.it; 2 Department of Pharmacy, University of Salerno, 84084 Fisciano, Italy; aambrosone@unisa.it (A.A.); aleone@unisa.it (A.L.); pdelgaudio@unisa.it (P.D.G.); 3 Institute for Sustainable Plant Protection, National Research Council, 80055 Portici, Italy; michelina.ruocco@ipsp.cnr.it; 4 MS Proteomics Research Group, Institute of Organic Chemistry, Research Centre for Natural Sciences, 1117 Budapest, Hungary; liliat7@gmail.com; 5 Institute of Biosciences and BioResources, Research Division Naples, National Research Council, 80131 Naples, Italy; ramesh.chem2008@gmail.com (R.B.); immacolata.fiume@ibbr.cnr.it (I.F.)

**Keywords:** root exudate, tomato, extracellular vesicles, plant-pathogen interactions, proteomics, fungal pathogens, biocargo, *Fusarium*, *Botrytis*, *Alternaria*

## Abstract

Extracellular Vesicles (EVs) play pivotal roles in cell-to-cell and inter-kingdom communication. Despite their relevant biological implications, the existence and role of plant EVs released into the environment has been unexplored. Herein, we purified round-shaped small vesicles (EVs) by differential ultracentrifugation of a sampling solution containing root exudates of hydroponically grown tomato plants. Biophysical analyses, by means of dynamic light scattering, microfluidic resistive pulse sensing and scanning electron microscopy, showed that the size of root-released EVs range in the nanometric scale (50–100 nm). Shot-gun proteomics of tomato EVs identified 179 unique proteins, several of which are known to be involved in plant-microbe interactions. In addition, the application of root-released EVs induced a significant inhibition of spore germination and of germination tube development of the plant pathogens *Fusarium oxysporum*, *Botrytis cinerea* and *Alternaria alternata.* Interestingly, these EVs contain several proteins involved in plant defense, suggesting that they could be new components of the plant innate immune system.

## 1. Introduction

Cells from all kingdoms of life secrete biomembrane-enclosed vesicles into the extracellular space. Extracellular vesicles (EVs) are a mix of heterogeneous populations of structures with a broad size distribution (30–1000 nm). Several lines of evidence indicate that EVs are involved in the horizontal transfer of bioactive cargos, such as proteins, nucleic acids (DNA, mRNA and microRNA), and other molecules including carbohydrates, lipids and secondary metabolites [1]. Owing to this characteristic, EVs have been reported to participate in cell-to-cell communications and in many physiological and physio-pathological processes, such as signal transduction, cell cycle, immune response, neurological diseases, inflammation and tumorigenesis [2].

Paramural vesicle structures between the plasma membrane and cell wall in plant tissues were firstly observed by transmission electron microscopy (TEM) more than half a century ago [3]. However, only recently, they have been isolated from apoplastic washing fluids of Arabidopsis leaves and sunflower seeds [4,5]. The role of apoplastic vesicles seems to be primarily linked to plant–pathogen interactions, inter-kingdom communication and stress response [6,7,8,9]. Proteomic analyses of apoplastic vesicles isolated from *A. thaliana* revealed an enrichment of proteins involved in biotic and abiotic stress responses [5]. Plant infected with *Pseudomonas syringae* or elicited with salicylic acid showed enhanced vesicle secretion [5]. Recently, plant apoplastic vesicles have been proved to carry also small non-coding RNAs, such as micro (miRNAs), short interfering RNAs (siRNAs) and a new class of “tiny RNAs” (10 to 17 nt), the functions of which are still to be elucidated [10]. Fascinatingly, small EVs were shown to transfer short RNAs from plant to pathogen cells that trigger a so-called host-induced gene silencing, a mechanism that allows the regulation of gene expression of the invading pathogen or parasite [7].

Nanometer-sized biomembrane vesicles have also been isolated from complex food matrices of plant origin, such as homogenized roots of ginger and carrots, and fruit juices of different citrus species and grapes [11,12,13]. It is worth mentioning that vesicles isolated from homogenized plant materials and fruit juices were shown to contain a complex mixture of intra- (transport vesicles, secretory vesicles, etc.) and extracellular vesicles. These new and innovative biomaterials are being exploited in their native forms due to their proven anti-inflammatory, anticancer and tissue regenerative properties as well as their action on the gut microbiome in vitro and in vivo [11,14,15]. Moreover, they can be used as nanovectors for the delivery of bioactive molecules [16]. 

Most of the current studies address the secretion, function and bioactivities of apoplastic vesicles that are localized in the gas- and water-filled plant intercellular space or of the more complex mixture of intra and extracellular vesicles isolated from edible plants. Here, we highlighted that EVs can also be released by root cells of tomato. These EVs are extramural as they pass the root cell walls and are released into the environment. We show that root EVs can inhibit spore growth of fungal pathogens and thus may have roles in inter-species communications and plant immunity.

## 2. Results and Discussion

### 2.1. Isolation and Characterization of Root-Derived EVs

**Figure 1:**
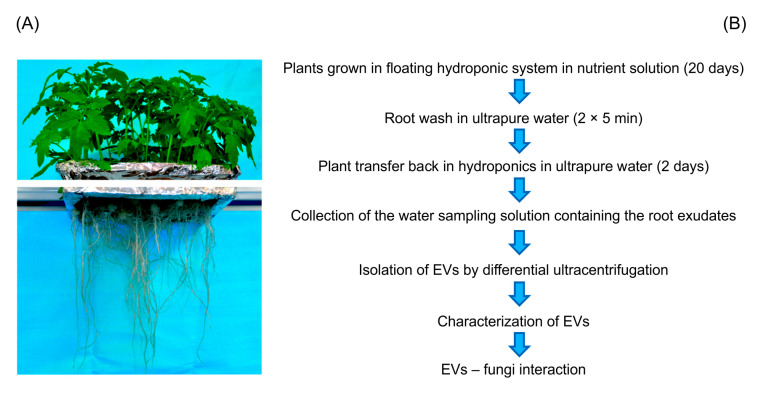
Experimental design used for collection and characterization of small Extracellular Vesicles (EVs) released by tomato roots. (**A**) Twenty-day-old tomato plants after removal from the nutrient solution and before transfer to ultrapure water for 2 days for the collection of root exudates (**B**) Schematization of the experimental design for plant growth, root exudate collection and EVs isolation and characterization.

EVs were isolated from a sampling solution of root exudates in ultrapure water of tomato (*Solanum lycopersicum* L.) plants cultivated in a hydroponic system, using membrane filtration followed by differential ultracentrifugation (dUC) as schematized in Figure 1. The yield of EVs obtained was low (30–50 μg of protein from 500 mL sampling solution, corresponding to approximately 1 μg of proteins/plant).

**Figure 2:**
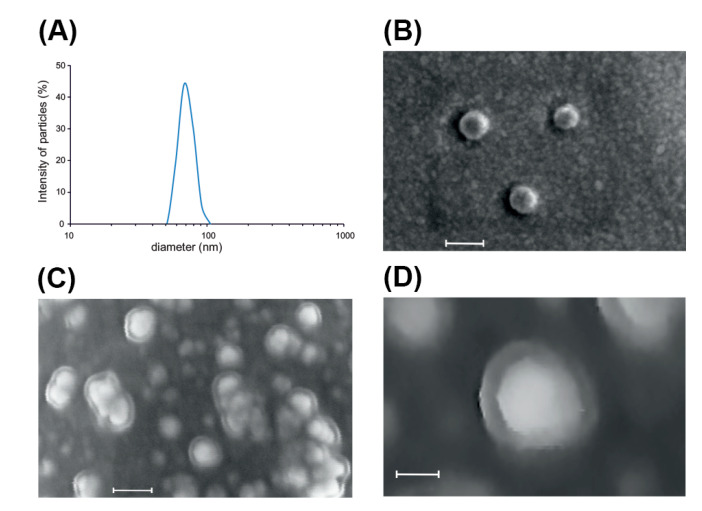
Physical characteristics of root-derived extracellular vesicles. (**A**) EVs size distribution curve obtained by dynamic light scattering (DLS). (**B**) Electron micrograph of three EVs. Scale bar, 100 nm. (**C**) Large view of density-sensitive backscattered electron imaging showing monodispersed EVs together with some aggregates. Scale bar, 100 nm. (**D**) Close-up view image of (**C**) showing the outer lipid layer with lower density with respect to the inner nano-sized structure. Scale bar, 30 nm.

Dynamic light scattering (DLS) showed a narrow size distribution of the EVs (40–100 nm), with an average diameter around 70 ± 5 nm (Figure 2A). Microfluidic resistive pulse sensing (MRPS) confirmed the distribution in the nm size range with an average peak diameter 51 nm (measured size range 50–300 nm) and measured the particle concentration as 6.00 × 10^10^ particles μg^−1^ of protein (Figure S1). Based on the hypothesis that contaminating protein in the samples should have a lowering effect on the particles-to-protein ratio [17], the high particles-to-protein ratios measured in our samples could indicate that the vesicle preparation was pure or contained relatively less proteins when compared to mammalian systems. The estimated EV yield per plant root system is 6.00 × 10^10^ particles/plant. The slight difference between the size measured by DLS and MRPS could be explained by the known bias of DLS toward larger particles in the intensity-weighted distribution, which can be noted when the DLS technique is used for the characterization of polydisperse samples [18]. EVs released by tomato roots appear smaller than the apoplastic vesicles isolated from *Vinca minor* (380 nm), *Viscum album* (280 nm), *Nicotiana tabacum* (70–520 nm) [19] and *Arabidopsis thaliana* (50–300 nm) [5]. We could not detect measurable amounts of other EVs populations (e.g., microvesicles) neither from the filtrate nor from the retentate in the low speed centrifugation step (15,000× *g*).

Secondary scanning electron microscope (SEM) analysis confirmed the presence of round-shaped nanostructures within the size range measured by DLS and MRPS (Figure 2B). Microstructural analysis conducted by backscattered electron imaging, which allows detecting material density differences, revealed that tomato root-derived EVs are formed by a low-density outer layer and a high-density inner compartment, resembling the typical lipid bilayer-enclosed nanostructures (Figure 2C,D).

### 2.2. Shot-Gun Proteomics of Root-Derived EVs

Protein composition of EVs released by tomato roots was analyzed by sodium dodecyl sulfate polyacrylamide gel electrophoresis (SDS-PAGE), which demonstrated the reproducibility of EVs isolation (Figure S2). Shot-gun proteomics identified 179 unique proteins with logprob values >3 in the two biological replicates (Table S1). There were 100 proteins commonly found in the two samples (Table S2). More than 70% of the commonly identified proteins have overlapping Cluster of Orthologous Groups (COG) or computed COG (ENOG) values (Table S2) with the EV proteins published in EVpedia [20], a database collecting EV data from different species. This indicates that the protein cargo of tomato root-derived EVs is highly similar to that reported for other eukaryotic and prokaryotic organisms. They include several proteins from the 14-3-3 protein family, actin, calmodulin, annexins, aquaporins, calreticulin and fatty acid binding proteins (Table S2) and support the hypothesis that biogenesis of EVs is evolutionarily conserved across the three kingdoms [21]. Table 1 reports the 20 top ranking proteins based on their intensities. Amongst them, we detected primary and secondary active transporters specific of plant cell membranes, such as plasmalemma and vacuolar H^+^-ATPases, five members of the aquaporin family, as well as nitrate and phosphate transporters (Table 1). The presence of plasma membrane transporters in the root EV preparations was expected as they are highly abundant in the plant plasma membrane. ATPases, for example, are abundant in vesicles isolated from fruits of different citrus species [13] but also in *Homo sapiens* EV samples [22]. Similarly, the expression of aquaporins, water channel proteins that are expressed in various membrane compartments of plant cells, is not surprising. Interestingly, aquaporins are also important constituents with diagnostic features of urinary EVs, as it has been shown by Oshikawa et al. [23].

To identify possible contamination from microorganisms, mass spectral peak lists from the collision-induced dissociation spectra that were not associated to proteins identified in the *S. lycopersicum* taxa were searched specifying all taxonomy. These analyses did not identify specific bacterial or fungal proteins that could count for heavy contamination.

Interestingly, tomato root-derived EVs and apoplastic vesicles isolated from Arabidopsis [5] share many similar proteins, such as family members of Casparian strip membrane domain protein (CASP)-like proteins, ATP-binding cassette (ABC) transporters, aquaporins, ammonium transporter 1, V-type proton ATPase subunits, protein NRT1/PTR FAMILY, patellin, vacuolar H^+^-ATPase A1 subunit, uclacyanin, t-SNARE proteins (SNAP), hypersensitive-induced response proteins. This finding, although preliminary, suggests a common origin of vesicles present in the plant apoplast with those released by roots that need to be further investigated.

**Figure 3:**
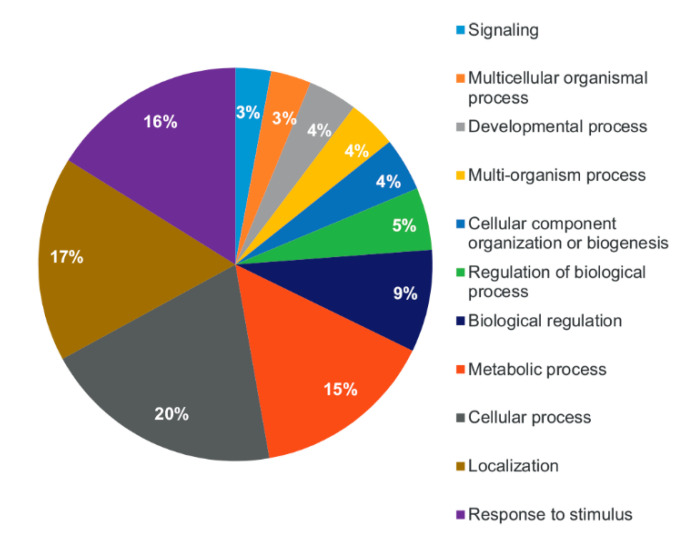
Gene Ontology analysis (Biological Process) of the proteome of tomato root-released EVs.

Gene Ontology (GO) analysis was used to group the 100 common EVs proteins according to biological process (BP) GO term. Most of the proteins belong to the wide categories “cellular process” (54 proteins), “localization” (46 proteins) and “response to stimulus” (44 proteins) (Figure 3). 

A high proportion of the identified proteins (23 out of 100) are known to participate in plant defense responses (Table 2). Several of them are involved in recognition and signal transduction, such as three receptor-like serine/threonine-protein kinases and two putative late blight resistance proteins (R1A-10 and R1A-3) [24,25]. The presence of these receptors, alongside with calmodulin 5 and NDR1/HIN1-like protein, associated to biotic stress signaling, may indicate that root-released EVs could participate in pathogen perception and activation of the immune response that restricts pathogen growth. Similarly, the identification of proteins involved in immune signaling in the Arabidopsis EVs leaf proteasome, such as RPM1-interacting protein 4, led to hypothesize a function of EVs in the signal cascade that modulates pathogen recognition [5]. Consistently with this role, the root-released EVs protein cargo also included proteins involved in a variety of defense mechanisms (Table 2), such as the hypersensitive-induced response protein 1, a germin-like protein (GLP) subfamily 1 member 19, and the monocopper oxidase-like protein SKU5, with a role in cell-wall reinforcement and expansion [26]. GLPs participate in cell wall reinforcement during the formation of callose-rich papillae and were reported to be crucial components of plant basal host resistance [27,28]. Moreover, the root-released EVs carried defense proteins such as an ethylene-responsive proteinase inhibitor 1, as well as two endochitinases and a glucan endo-1,3-beta-glucosidase B precursor, the latter two protein classes being able to decompose pathogen cell walls [29,30].

The presence of such a defense toolkit in the EVs released by roots suggests that, similarly to apoplastic vesicles [9], they can transport a plethora of defense-related proteins and shuttle this arsenal outside the plant. We may speculate that, in the absence of a pathogen challenge, these nanostructures may create a preventive pathogen-free zone for optimal root growth and/or deploy a defense arsenal in proximity of potential access points for soil pathogens (e.g., injured roots).

### 2.3. Bioactivity of Root-Derived EVs on Plant Pathogens

**Figure 4:**
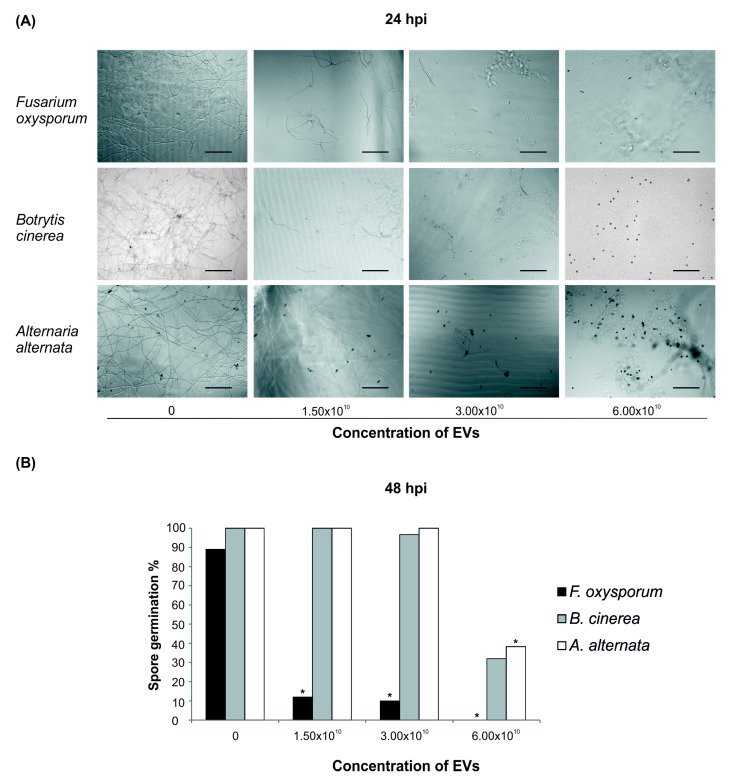
Tomato root-derived EVs have antifungal activity against plant pathogens. (**A**) Inverted microscope micrographs (20× magnification, Leica DMi8) of *Fusarium oxysporum*, *Botrytis cinerea* and *Alternaria alternata* spore germination at 24 h post inoculation (hpi) with increasing concentrations of the EVs. Scale bar, 50 μm. (**B**) Average spore germination (*n* = 3) (%) for *F. oxysporum, B. cinerea* and *A. alternata* at 48 hpi of treatment with increasing concentrations of the EVs. EV resuspension buffer was added to the growth medium of spores of the control sample. Results are representative of three independent experiments. * indicate significance with *p <* 0.05 by using one way ANOVA Tukey post hoc test.

To address this last hypothesis, spores of the tomato soil-borne pathogen *Fusarium oxysporum*, and of the tomato air-borne pathogens *Botrytis cinerea* and *Alternaria alternata* were incubated with tomato root-released EVs. Spore germination of all tested fungi was affected when EVs were added to the substrate (Figure 4A,B). Spore germination of *F. oxysporum* was the most affected by the presence of EVs in the germination solution, since 48 h post inoculation (hpi) only 12% spores germinated in a solution containing 1.50 × 10^10^ EVs (Figure 4B). In contrast, at the same EVs concentration, almost 100% spores of *A. alternata* and *B. cinerea* germinated, while a strong inhibition was observed only at the highest concentration (6.00 × 10^10^ EVs, Figure 4B). It is worth noting that, although *B. cinerea* and *A. alternata* spore germination at 48 hpi was close to 100% in the presence of up to 3.00 × 10^10^ EVs (Figure 4B) and that the development of germination tube was progressively inhibited by increasing EVs concentrations, as indicated by the corresponding decrease in tube length at 24 hpi (Figure 4A).

It is well known that roots can modulate the function and the structure of the rhizobiome through the secretion of molecules that can stimulate or repress microorganisms activity driving to healthy or diseased plants. A range of chemical compounds have been found in root exudates that have been reported to act as repellents or chemoattractants of soil microbes, during both pathogenic and beneficial interactions. Recently, Li et al. [31] reported the inhibitory activity of wheat root exudates against the pathogenic fungus *F. oxysporum f. sp. niveum*. Similarly, it is largely accepted that the plant mechanisms leading to the attraction of different beneficial microbes are active, directional and expressed systemically in the plant [32]. Within the array of mechanisms evolved by plants against biotic stresses, EVs can function as a defense system [9]. Most proteins identified in the tomato root EVs proteome, including endochitinases, an endoglucanase and a trypsin inhibitor, have been correlated with pathogenesis. Chitinases play fundamental roles not only in plant metabolism but also in the response to biotic and abiotic stresses, and have been classified in four families of the recognized pathogenesis-related proteins [33]. Moreover, the root EVs proteome contained proteins involved in oxidative stress response such as annexin p34 [34], which contributes to resistance against *Phytophthora infestans* in the potato apoplast [35]. We also detected proteins participating in general plant immunity such as calmodulin, implicated in calcium-mediated signal transduction, and patatin-like protein 2 (Table 2), which were both reported to regulate the response against *B. cinerea* infection in tomato and Arabidopsis [36,37].

To the best of our knowledge, this is the first study reporting the isolation and the proteomic biocargo characterization of root EVs secreted in the environment. The presence of a high number of proteins involved in the perception and transduction of plant–pathogen interactions as well as of typical defense proteins allows us to speculate that the protein cargo of tomato root-derived EVs may account, at least in part, for the demonstrated ability of the EVs to inhibit spore germination and germination tube development of different pathogens *F. oxysporum*, *B. cinerea* and *A. alternata*. RNA-mediated gene silencing could also contribute to this inhibition, as it was demonstrated for apoplastic EVs [7].

## 3. Conclusions

Besides contributing to the advancement of EV knowledge in plants, the results presented here reveal for the first time that plant roots release nano-sized vesicles with a narrow size distribution and round shape into the environment. EVs released by tomato roots carry homologs of proteins typically present in mammalian-derived extracellular vesicles and in plant-derived apoplastic vesicles. The finding that EVs are secreted by the root in the absence of overt infections suggests that they may have a physiological function in the plant immune system. Indeed, here we show their bioactivity against fungal pathogens in vitro. Further efforts are needed to unveil the mechanisms of EV release from root cells, largely unknown in plants, and to describe the interactions between EVs and soil microbes. Possible changes in proteome composition of root-secreted EVs in pathogen-challenged tomato plants are also worth studying, in order to establish their contribution to plant disease resistance.

## 4. Materials and Methods 

### 4.1. Plant Growth and Root Exudate Sampling Strategy

Seeds of tomato (*Solanum lycopersicum* cv. ‘Crovarese’) kindly provided by La Semiorto Sementi s.r.l. (Lavorate di Sarno, Italy) were surface sterilized with 50% (*v*/*v*) of commercial bleach solution (final concentration 1.5% sodium hypochlorite) for 20 min, washed in sterile distilled water, and then germinated in Petri dishes on sterile filter paper in the dark in a growth chamber at 25 °C. Four-day germinated seedlings were transferred into dark plastic boxes (30 plants/tray) containing a nutrient solution, previously described in De Palma et al. [38], which was refreshed weekly. Plants were maintained in a hydroponic floating system in a walk-in growth chamber at 25/21 °C (day/night), 65% relative humidity, 15:9 h light:dark photoperiod. After 20-days, plants were removed from the nutrient solution and the roots were carefully washed two times for 5 min in ultrapure water (Milli-Q UF Plus, Millipore, Molsheim, France). Plants were then transferred back into the hydroponic floating system where the nutrient solution was substituted with 1000 mL ultrapure water (Milli-Q UF Plus, Millipore, Molsheim, France) for each tray of 30 plants. After 48 h of further growth in the growth chamber in the above-indicated conditions (25/21 °C, 65% relative humidity, 15:9 h light:dark photoperiod), the whole of the remaining water sampling solution (approximately 500 mL) was collected and used for EVs isolation. Six independent experiments were conducted each with two biological replicates.

### 4.2. Isolation of Extracellular Vesicles

Extracellular vesicles were isolated from the sampling solution (volume approximately 500 mL) using a differential centrifugation method [39]. Briefly, protease inhibitors (250 μL 1 mg mL^−1^ leupeptine, 1.25 mL 100 mM phenylmethylsulfonyl fluoride and 800 μL 1 M sodium azide) were added and the solution was filtered using 0.22 μm pore size membrane filters. Low velocity centrifugation was performed using 50 mL Beckman polypropylene tubes in a JA-25.50 Beckman (Beckman Coulter Inc., Brea, CA, USA) fixed angle rotor at 15,000× *g* for 20 min at room temperature. No visible pellet was observed after this step. Supernatant was subjected to ultracentrifugation using polycarbonate tubes (Beckman, 26 mL) in a Beckman Type 70Ti rotor at 150,000× *g* for 120 min at 4 °C. Pellet was re-suspended in 50 μL 10 mM Tris-HCL pH 8.6. Protein concentration was measured by micro BCA assay (Thermo Fisher Scientific Inc., Rockford, IL, USA) using Nanodrop 2000 (Thermo Fisher Scientific Inc., Waltham, MA, USA). The EV quantities obtained were insufficient to perform further gradient ultracentrifugation-based purification steps.

### 4.3. Protein Profiling by SDS-PAGE

The quality of the vesicle samples and the reproducibility of EVs isolation was controlled using sodium dodecyl sulfate polyacrylamide gel electrophoresis (SDS-PAGE). Samples (20 μg protein based on micro BCA measurement) were electrophoretically separated under reducing conditions on a precast Novex Bolt 4–12% Bis-Tris Plus gel, using Bolt MOPS SDS running buffer (Thermo Fisher Scientific Inc., Waltham, MA, USA), according to the manufacturer’s instructions and stained with colloidal Coomassie blue.

### 4.4. Liquid Chromatography-Electrospray Ionization Mass Septrometry

EV isolates were lysed using 5 freeze-thaw cycles in the presence of Rapigest (Waters, Milford, MA, USA) detergent according to the manufacturer recommendations. Lysed vesicles were in-solution digested using Trypsin (Mass Spec grade, Promega Corporation, Madison, WI, USA) at a 1:100 protein:enzyme ratio. One µg tryptic digest was analyzed on a Dionex Ultimate 3000 nanoRSLC (Dionex) coupled to a Bruker Maxis II mass spectrometer (Bruker Daltonics GmbH, Bremen, Germany), via Captive Spray nanobooster ion source. A total of 1 μg of digested proteins was injected on an Acclaim PepMap100 C-18 trap column (100 µm × 20 mm, Thermo Fisher Scientific Inc., Waltham, MA, USA) using 0.1% TFA for 8 min at a flow rate of 5 µL min^−1^. Peptide separation was achieved on an ACQUITY UPLC M-Class Peptide BEH C18 column (130 Å, 1.7 µm, 75 µm × 250 mm; Waters, Milford, MA, USA) under the following conditions: 300 nL min^−1^ flow rate, 48 °C column temperature, gradient method from 4% B to 50% B in 90 min. Solvent A was water + 0.1% formic acid, solvent B was acetonitrile + 0.1% formic acid. Data dependent analysis was performed using a fixed cycle time of 2.5 s. MS spectra were acquired at 3 Hz, while MS/MS spectra at 4 or 16 Hz depending on the intensity of the precursor. Singly charged ions were excluded from the analysis. Raw data files were processed using the Compass Data Analysis software (Bruker Daltonics GmbH, Bremen, Germany). Protein identification was performed using the Byonic software, version 2.15.7 (Protein Metrics Inc., Cupertino, CA, USA). Protein sequences were searched against the NCBI *S. lycopersicum* database (45231 entries) using the following criteria: 10 ppm precursor mass tolerance, 50 ppm fragment mass tolerance. Two miss cleavages were allowed and semi specific N-ragged digestion specificity. Carbamidomethylation on cysteines was set as fixed modification and the following variable modifications were applied: dicarbamidomethylation (N-term), deamidation (N), pyro-Glu (Q, E), ammonia-loss (N-term C), carbamylation (M) and acetylation (protein N-term). MaxQuant software version 1.5.3.30 [40] was used for label free quantitation.

### 4.5. Bioinformatics

The detailed experimental protocol and EVs characteristics were deposited in EV-Track [41] with EV track ID: EV190052. Proteomics data were deposited in Vesiclepedia public database [42]. Functional annotation of the protein data was performed by using Blast2Go software v.5.2.5. Identified proteins in FASTA format was used as input data. The Blastpfast search algorithm was used via NCBI web service using taxonomy filter green plants (taxa: 33090, Viridiplantae), number of blast hits 20 and expectation value 1.0 × 10^−3^. The InterPro domain searches were performed using the input FASTA files. The Blast hits of each protein sequence were mapped with Gene Ontology (GO) terms deposited in the GO database. Enzyme Code (EC) and Kyoto Encyclopedia of Genes and Genomes (KEGG) annotations were performed on hits resulted in GO annotation. Orthology assignment and clusters for orthologous groups (COG) annotation were performed by EggNOg Mapper version 5.4.1 [43]. EggNOG OGs were used to compare protein datasets between different taxa and EVpedia [20] deposited OGs. Selection of defense-related proteins was manually curated.

### 4.6. Dynamic Light Scattering (DLS)

EVs size distribution was measured by dynamic light scattering (DLS) using a Zetasizer Ver. 7.01, Malvern Instrument (Malvern, Worcestershire, UK). A total of 1 μg of EVs was dispersed in deionized water and the intensity of the scattered light was measured with a detector at 90° angle at room temperature. Measurements were carried out in biological triplicate.

### 4.7. Microfluidic Resistive Pulse Sensing (MRPS)

EVs concentration and size distribution were measured by microfluidic resistive pulse sensing (MRPS) using an nCS1 instrument (Spectradyne LLC, Torrance, CA, USA). Samples from one representative experiment, containing 0.18 μg μl^−1^ protein, were diluted 100× in 1× PBS + 1% Tween 20 (PBST) and 3 μL were loaded into a TS300 cartridge.

### 4.8. Scanning Electron Microscopy (SEM)

Extracellular vesicles were diluted to 1 μg μL^−1^ concentration in ultrapure water (Milli-Q UF Plus, Millipore, Molsheim, France), fixed in 4% paraformaldehyde and spotted onto carbon-coated grids. SEM images of the samples were then acquired using a Tescan S8000 microscope equipped with secondary electron and backscattered electron detectors (TESCAN, Brno, Czech Republic). Analyses were conducted both at 1.5 and 5 keV without any coating of the particles, respectively.

### 4.9. Preparation of Fungal Spore Suspension

Spore suspensions were obtained from *Botrytis cinerea* SC1, *Alternaria alternata* A1 and *Fusarium oxysporum* Zaf1. All fungi belong to the collection of the Institute for Sustainable Plant Protection. Spore suspensions of each plant pathogen were obtained from −80 °C stocks, inoculated on Petri dishes containing Potato Dextrose Agar (Difco) and grown at 25 °C until a complete sporulation occurred. Spores from each pathogen were recovered from Petri dishes by scraping the surface of the growing colonies with a sterile spatula and transferred into 15 mL of sterile distilled water. Concentration of the obtained spore suspensions was adjusted to 1.50 × 10^5^ mL^−1^ for subsequent analysis.

### 4.10. Vesicle/Spore Interaction Tests

To investigate the activity of root derived EVs on spore germination of three different phyto-pathogens, germination tests were performed in 96 wells flat-bottom tissue culture plates at 25 °C. Pathogen spores were incubated in Potato Dextrose Broth (PDB; Difco) in the presence of 6.00 × 10^10^, 3.00 × 10^10^ and 1.50 × 10^10^ EVs particles. Assay mixtures (100 μL volume) contained: 10 μL of a spore suspension (1.00 × 10^5^ mL^−1^) of each tested fungus (*B. cinerea* or *A. alternata* or *F. oxysporum*), 30 μL of 1× PDB, 6 μL of EVs suspension and 54 μL of distilled sterile water. Controls contained 6 μL of 10 mM Tris-HCL pH 8.6 (EVs resuspension buffer) instead of vesicle suspension. Three technical replicates per assay were performed, and the experiment was repeated three times. Spore germination was evaluated using an inverted DMi8 microscope (Leica, Wetzlar, Germany) after 24 and 48 h, recoding the number of germinated spores and taking representative photos for each tested fungal pathogen.

## Figures and Tables

**Table 1:** List of the 20 most abundant proteins within the dataset of the 100 most common proteins between two biological replicates, as determined by quantitative label-free proteomics.

No.	Protein GI	Name
1	1022943236	H(+)-ATPase 4
2	1104626054	hypersensitive-induced response protein 1
3	726965338	probable aquaporin PIP2-1
4	350534408	Plasma membrane intrinsic protein 1
5	1104594347	12S seed storage protein Cruciferin D (CRD)
6	723685072	senescence-induced receptor-like serine/threonine-protein kinase
7	723688012	high-affinity nitrate transporter 3.2-like
8	1104611050	patatin-like protein 2
9	921274020	vacuolar H+-ATPase A1 subunit isoform
10	460370435	V-type proton ATPase subunit B 2
11	723754262	ferric reduction oxidase 4
12	460376506	phosphate transporter
13	460404122	uncharacterized protein LOC101257107
14	460401047	polyphenol oxidase E, chloroplastic-like
15	1104638510	NDR1/HIN1-Like protein 3-like isoform X2
16	927928759	aquaporin PIP2-7-like
17	350537435	glucan endo-1,3-beta-glucosidase B precursor
18	1114439811	probable aquaporin PIP-type pTOM75
19	10263446685	calmodulin 1
20	902763248	putative PIP-type aquaporin

**Table 2:** Plant defense-related proteins identified in EVs released by tomato roots.

Protein GI	Name
460406368	endochitinase 4
1104611050	patatin-like protein 2
350537435	glucan endo-1,3-beta-glucosidase B precursor
1104626054	hypersensitive-induced response protein 1
1026346657	calmodulin 5
460408499	trypsin inhibitor 1-like
460403785	probable linoleate 9S-lipoxygenase 5
350538805	annexin p34
460370493	lysM domain-containing GPI-anchored protein 2
124192	ethylene-responsive proteinase inhibitor 1
723690477	putative late blight resistance protein homolog R1A-10
460382464	putative late blight resistance protein homolog R1A-3
1104638510	NDR1/HIN1-like protein 3-like isoform X2
460409616	putative LRR receptor-like serine/threonine protein kinase At4g00960
723690472	putative late blight resistance protein homolog R1A-3
544011	basic 30 kDa endochitinase
460404505	germin-like protein subfamily 1 member 19
460382045	CASP-like protein PIMP1
460408543	probable LRR receptor-like serine/threonine-protein kinase At1g06840
723687659	hypersensitive-induced response protein 1
460400431	monocopper oxidase-like protein SKU5
350537023	wound/stress protein precursor
1143632856	MRLK1 serine/threonine protein kinase, partial

## Supplementary Materials

The following are available online at https://www.mdpi.com/2223-7747/9/12/1777/s1, Figure S1: Particle size distribution and concentration of EVs released by tomato roots. Microfluidic resistive pulse sensing (MRPS) analysis was performed by nCS1 (Spectradyne) using a TS-300 single-use disposable cartridges that allows for particle size analysis in the range from 50 to 300 nm in diameter. Sample from one representative experiment, containing 0.18 μg μL^−1^ proteins, as measured by BCA assay, was diluted hundred times in a buffer containing PBS 1% (*v*/*v*) Tween 20. Three microliters from the sample were processed for MRPS Spectradyne measurements using a TS300 cartridge. “Total stock concentration” (1.08 × 10^10^ p/mL) was calculated based on the integration of the distribution peak observed in the stock solution (0.18 μg μL^−1^). Based on this measurement, the ratio of particles to protein is 6 × 10^10^ EVs/μg. Line in the middle shows the values observed at full width at half maximum (FWHM). N is the number of particles counted; Figure S2: SDS-PAGE analysis of EVs samples released by tomato roots. The gel image shows similarities between the protein profiles of two biological replicates from one representative experiment; Table S1: Proteomic analysis of the tomato root EVs. Worksheet “EXPERIMENTAL DETAILS” shows the details relative to the two biological replicates. For data analysis, only the 179 proteins with logprob >3 values were considered. Worksheet “PROTEIN DETAILS” shows the protein data obtained for the two samples; experiment 1 is relative to sample 1 and experiment 2 is relative to sample2. Identified proteins are ranked by log prob values. Proteins identified by a log prob value < 3 are highlighted in yellow and were not considered in the data analysis. “Total intensity” is the intensity value associated to each protein in the label free quantitation using MaxQuant software version 1.5.3.30; “# AA’s in protein” shows the number of amino acids in the protein; “Protein DB number” refers to the protein data bank number; “Coverage %” is the percent coverage calculated by dividing the number of amino acids in all found peptides by the total number of amino acids in the entire protein sequence; “# of unique peptides” shows the number of unique peptides identified and “# of mod peptides” shows the number of modified peptides identified; “Best score” refers to the score obtained by the Byonic software. Worksheet “PEPTIDE DETAILS” shows the peptide data obtained from MaxQuant-based proteomic analysis of the two biological replicates (experiment 1 and experiment 2). “Entrez Gene ID” shows the identifier of the gene in the NCBI data base used for protein identification; “mz” is the mass to charge ratio of the precursor ion selected for the analysis; “z” is the number of charges; “Peptide score” is the score obtained in the Byonic search; ”peptide start” is the number of amino acid in the sequence of the protein where the peptide sequenced start; “residue before” and “residue after” are the amino acid residues before and after the peptide start in the protein sequence, respectively; “Sequence identifier Protein DB” is the identifier of the protein in the Protein data base; Table S2: Detailed annotation of the tomato root-derived EVs proteins. COGs and ENOG accession numbers determined by EggNOG mapper of the 179 proteins identified in the EVs released by tomato roots, compared to EVpedia proteins. Gene identifiers, and Gene Ontology analysis against different databases are also reported. KEGG Onthology categories (KO) and Biochemical, Genetic and Genomic (BiGG) reactions are indicated. “Total intensity” is the intensity value associated to each protein in the label free quantitation using MaxQuant software version 1.5.3.30. The 100 proteins common to the two biological replicates are highlighted in green.
