# Supplementary material for: Plant Roots Release Small Extracellular Vesicles with Antifungal Activity
Source: Plants (Basel). 2020 Dec 15;9(12):1777. doi: 10.3390/plants9121777 (PMC7765200; doi:10.3390/plants9121777)
Supplement: Supplementary file 1 [file plants-09-01777-s001.zip › Supplementary files REV1/De Palma, Ambrosone_Supplementary Figures_REV1.pdf]

Plant roots release small extracellular vesicles with antifungal activity

Monica De Palma, Alfredo Ambrosone, Antonietta Leone, Pasquale Del Gaudio, Michelina Ruocco, Lilla Turiák, Ramesh Bokka, Immacolata Fiume, Marina Tucci and Gabriella Pocsfalvi

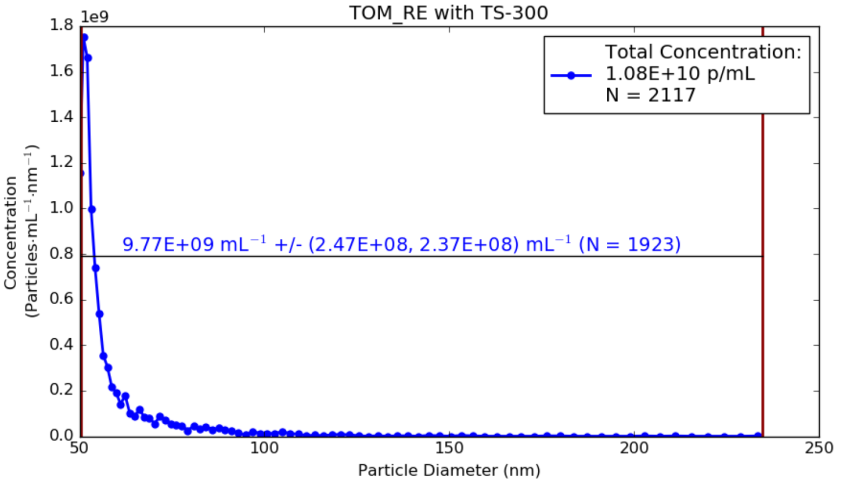

Sample	Peak Diameter	Measured Size Range	Stock Concentration (particles/mL)	Dil. factor
TOM_RE	51 nm	50 – 300 nm	1.08×10^{10}	100x

Figure S1. Particle size distribution and concentration of EVs released by tomato roots. Particle size distribution and concentration of EVs released by tomato roots. Microfluidic resistive pulse sensing (MRPS) analysis was performed by nCS1 (Spectradyne) using a TS-300 single-use disposable cartridges that allows for particle size analysis in the range from 50 to 300 nm in diameter. Sample from one representative experiment, containing 0.18 µg μl^{-1} proteins, as measured by BCA assay, was diluted hundred times in a buffer containing PBS 1% (v/v) Tween 20. Three microliters from the sample were processed for MRPS Spectradyne measurements using a TS300 cartridge. “Total stock concentration” (1.08×10^{10} p/mL) was calculated based on the integration of the distribution peak observed in the stock solution (0.18 µg μl^{-1}). Based on this measurement, the ratio of particles to protein is 6×10^{10} EVs/µg. Line in the middle shows the values observed at full width at half maximum (FWHM). N is the number of particles counted.

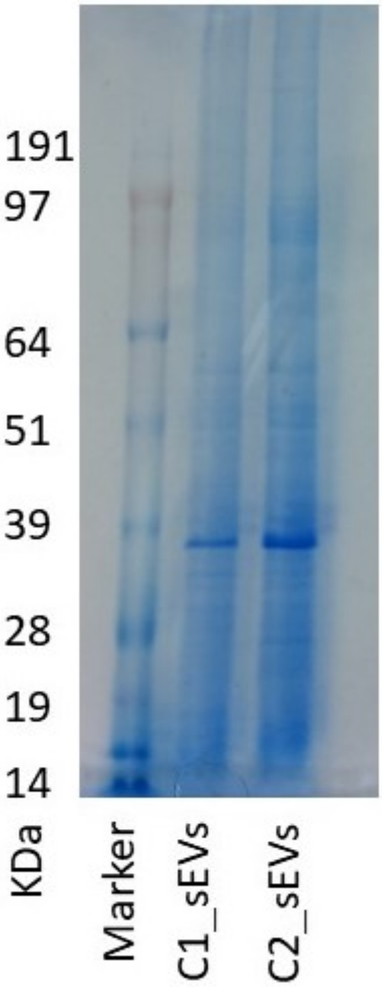

Figure S2. SDS-PAGE analysis of EVs samples released by tomato roots. The gel image shows similarities between the protein profiles of two biological replicates from one representative experiment.
